# Prospects on the Use of *Schizochytrium* sp. to Develop Oral Vaccines

**DOI:** 10.3389/fmicb.2018.02506

**Published:** 2018-10-25

**Authors:** Abel Ramos-Vega, Sergio Rosales-Mendoza, Bernardo Bañuelos-Hernández, Carlos Angulo

**Affiliations:** ^1^Grupo de Inmunología and Vacunología, Centro de Investigaciones Biológicas del Noroeste, La Paz, Mexico; ^2^Laboratorio de Biofarmacéuticos Recombinantes, Facultad de Ciencias Químicas, Universidad Autónoma de San Luis Potosí, San Luis Potosí, Mexico; ^3^Sección de Biotecnología, Centro de Investigación en Ciencias de la Salud y Biomedicina, Universidad Autónoma de San Luis Potosí, San Luis Potosí, Mexico; ^4^Escuela de Agronomía y Veterinaria, Universidad De La Salle Bajío, León, México

**Keywords:** adjuvant, bioencapsulation, microalgae, oral vaccine, thermostable vaccine, Algevir system

## Abstract

Although oral subunit vaccines are highly relevant in the fight against widespread diseases, their high cost, safety and proper immunogenicity are attributes that have yet to be addressed in many cases and thus these limitations should be considered in the development of new oral vaccines. Prominent examples of new platforms proposed to address these limitations are plant cells and microalgae. *Schizochytrium sp*. constitutes an attractive expression host for vaccine production because of its high biosynthetic capacity, fast growth in low cost culture media, and the availability of processes for industrial scale production. In addition, whole *Schizochytrium sp*. cells may serve as delivery vectors; especially for oral vaccines since *Schizochytrium sp*. is safe for oral consumption, produces immunomodulatory compounds, and may provide bioencapsulation to the antigen, thus increasing its bioavailability. Remarkably, *Schizochytrium sp*. was recently used for the production of a highly immunoprotective influenza vaccine. Moreover, an efficient method for transient expression of antigens based on viral vectors and *Schizochytrium sp*. as host has been recently developed. In this review, the potential of *Schizochytrium sp*. in vaccinology is placed in perspective, with emphasis on its use as an attractive oral vaccination vehicle.

## Relevance and challenges in oral vaccine development

Vaccination is a primary intervention against infectious diseases, thus, affordable vaccination campaigns for government budgets, especially in developing countries, are a priority. However, many potentially vaccine-preventable diseases in low-income countries are inadequately prevented due to an insufficient use of the existing vaccines. For instance, there is a lack of efficient distribution and delivery logistics, in addition to the associated high cost of the vaccine (Kochhar et al., 2013; Chen et al., 2014a). Most of the current available vaccines are designed for subcutaneous or intramuscular administration. However, these routes have limitations such as the problems associated with unsafe injections that are consequence of low economic resources and limited trained personnel (Hauri et al., 2004; Wilkhu et al., 2011). In addition, parenteral vaccines generally require “cold chain,” which represents further economic and logistical burdens (Kumru et al., 2014). Therefore, oral immunization with thermostable vaccines is highly desired since it avoids the need of cold chain, specialized devices, and trained personnel for administration (Scherliess, 2011).

Since the vast majority of pathogens infect their host through the mucosa, local immune responses at these sites serve as the first line of defense against the pathogen (Hornef, 2015). Interestingly, several vaccines administered via mucosal routes have a proven effective induction of both systemic and local immunity (Lamichhane et al., 2014). However, to achieve efficacy, higher and more frequently administered doses are required in oral immunization schemes when compared to intramuscular or subcutaneous vaccines; which is a consequence of antigen dilution and degradation in the gastrointestinal tract as well as a poor antigen uptake (Doherty, 2015; Truong-Le et al., 2015).

Another important aspect is related to the fact that the mucosal tissues maintain homeostasis by mounting specialized anti-inflammatory immune defenses, including the induction of tolerance against innocuous soluble substances and commensal bacteria (Kweon, 2014). Therefore, oral vaccination must overcome the induction of local and systemic immunological tolerance, known as oral tolerance (Wilkhu et al., 2011). This obstacle can be overridden by antigen encapsulation (Kai and Chi, 2008) and the inclusion of adjuvants to enhance the immunogenic properties of the formulation (Hasegawa et al., 2015; Savelkoul et al., 2015). Although several technologies for antigen encapsulation (Trovato and Berardinis, 2015) and adjuvants are under evaluation (Newsted et al., 2015), there is a clear need to continue in the exploration of new oral vaccination strategies; which is reflected by the limited number of oral vaccines available in the clinic (Yuki and Kiyono, 2009).

## Microalgae-made vaccines

Among the current trends in biotechnology for the production of biopharmaceuticals in attractive platforms, algae have been used to produce monoclonal antibodies, vaccine antigens, therapeutic enzymes, blood proteins, cytokines, growth factors, and growth hormones. Microalgae-based expression systems are inherently faster to develop, potentially less expensive, and require less space for production. In addition, the biomass is relatively inexpensive to produce. Algae-based vaccines offer antigen protection from proteolytic degradation due to the cell wall. In addition, subcellular compartmentalization may also influence antigen release and thus bioavailability (Gregory et al., 2013). Moreover, algae are capable of performing post-translational modifications (e.g., glycosylation in endoplasmic reticulum and Golgi) that are frequently important for the antigen activity, can be produced relatively fast and some species could be used to formulate vaccines in a straightforward manner since they hold a GRAS status (Specht and Mayfield, 2014). This notion has been primarily explored for the freshwater microalga *Chlamydomonas reinhardtii* with important advances toward the development of low cost orally-delivered vaccines (Bañuelos-Hernández et al., 2015; Rasala and Mayfield, 2015; Dyo and Purton, 2018). Two interesting reports in this area comprise a developed *S. aureus* vaccine based on *C. reinhardtii*, showing antigen yields up to 0.7% TSP. The vaccine was stable at room temperature up to 20 months. Moreover, the mucosal IgA and systemic IgG responses were induced in orally immunized mice subjected to a scheme consisting of priming and 4 boosts administered weekly. Remarkably, an 80% survival rate after a lethal challenge with *S. aureus* was achieved (Dreesen et al., 2010). In the same microalga, a vaccine against malaria was developed. The vaccine was able to induce the systemic IgG responses and conferred protection against *Plasmodium berghei* in terms of reduction of parasitic load in red blood cells from mice treated with a single vaccine dose (Dauvillée et al., 2010). Another case of an oral algae-based vaccine against malaria consisted of a fusion protein comprising the cholera toxin B subunit (CTB) as adjuvant and the antigen of *Plasmodium falciparum* Pfs25. In this case, the oral vaccination of BALB/c mice using algae producing CTB-Pfs25 elicited CTB-specific serum IgG, fecal IgA antibodies, as well as Pfs25-specific IgA antibodies (Gregory et al., 2013). Diatoms have also been applied for the expression of vaccine antigens with promising findings on the expression of IbpA DR2 antigen from *Histophilus somni* (Corbeil et al., 2015; Davis et al., 2017). Although no clinical trials are ongoing for algae-based vaccines, the technology seems promising and these evaluations could begin in the short term (Rosales-Mendoza and Salazar-González, 2014).

## Application of marine microalgae in vaccine development

Marine organisms are attractive hosts in this field as they are currently produced at industrial levels in culture media based on marine water to produce compounds with pharmaceutical, nutrition, and health applications; among other industrial applications (Mayer et al., 2011; Dewapriya and Kim, 2014). Interestingly, marine microalgae have been used in the production of vaccines. For instance, *Phaeodactylum tricornutum* was used to produce a monoclonal human IgG antibody against the Hepatitis B surface antigen (HBsAg) as well as HBsAg fused to GFP or an ER retention signal. The achieved antibody production was 8.7% of the total soluble protein (TSP; 1.6 mg per liter of culture or 21 mg antibody per gram algal dry weight), whereas HBsAg yields were up to 0.7% TSP (Hempel et al., 2011). Similarly, *Dunaliella salina* was transformed for the expression of HBsAg. In this case, the yields obtained were up to 3 ng/mg soluble protein and the positive clones were grown in non-selective liquid media for at least 60 generations; showing that the HBsAg protein was stably expressed in the transformed cells (Geng et al., 2003). On the other hand, the expression of the viral protein 28 (VP28) from the *White spot syndrome virus* was reported in the marine microalga *Dunaliella salina* with yields up to 780 μg VP28 per liter of culture. This vaccine was able to induce a 41% reduction in shrimp mortality after a lethal challenge experiment in orally immunized animals (Feng et al., 2014).

## Relevant characteristics of *Schizochytrium sp*. for vaccine development

**Figure 1:**
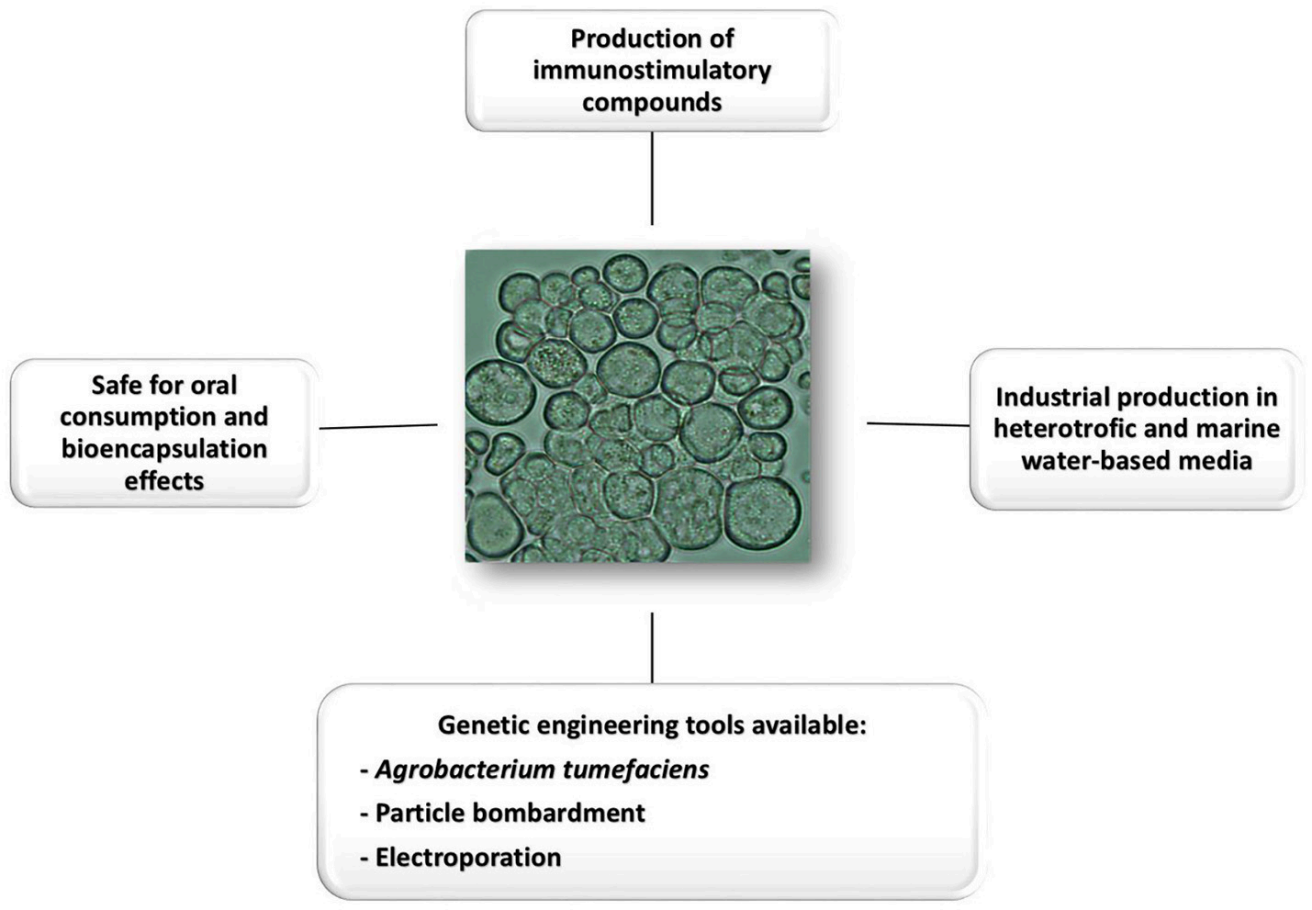
Compilation of the main attributes that make *Schizochytrium* an attractive host for vaccine production and oral delivery.

*Schizochytrium sp*., a thraustochytrid, is a heterokont marine microalgae with a cell diameter of about 9–14 μm belonging to the Labyrinthulomycetes class, which is used to produce Docosahexaenoic acid (DHA) that accumulates up to 50% of dry weight lipids. Furthermore, it contains up to 10% of protein and 25% of carbohydrates (Qu et al., 2013; Fedorova-Dahms et al., 2014; Yao et al., 2015). In addition β-carotene is accumulated at significant levels in some species of this genus (Aki et al., 2003; Yokoyama and Honda, 2007; Ren et al., 2010). *Schizochytrium sp*. can be propagated at the industrial scale in heterotrophic conditions in which low cost medium is used and no complex photobioreactors are required since the process does not depend on light irradiation. Since some species of *Schizochytrium sp*. may grow in marine water-based culture media, their industrial use could not interfere with fresh water sources used for agriculture (Barclay, 1992; Chang et al., 2014). This microalga is currently used as food supplement in mammals and poultry (Meale et al., 2014). For instance, the *Schizochytrium sp*. supplementation of the laying hen diet has a beneficial effect on egg production, egg weight, yolk color, and blood lipid profiles of the layer hen (Park et al., 2015). In addition, DHA from *Schizochytrium sp*. is a key component in dietary supplements, cosmetics products, and pharmaceutical formulations (Fedorova-Dahms et al., 2014; Aasen et al., 2016). Moreover, several immunomodulating compounds are also produced by this microalga, which highlights a potential contribution to vaccine efficacy when used as a delivery vehicle (Figure 1). For instance, it produces squalene, a compound with adjuvant activity (Hoang et al., 2014).

*Schizochytrium* sp. is an interesting alternative for vaccine production and delivery due to its capacity for recombinant protein expression, being able to efficiently export proteins toward the extracellular compartment; which is a substantial advantage over the bacterial hosts since the recombinant protein can be easily purified due to the simpler composition of culture supernatants. In addition, a singular advantage of *Schizochytrium sp*. is the capability of exporting full-length complex membrane-bound insoluble proteins in a secreted form while retaining full functionality and a properly active structure (Bayne et al., 2013). Since *Schizochytrium sp*. has singular properties among the algae species, the implications of this species in vaccinology are analyzed.

### Bioencapsulation effects

Since a major challenge in oral vaccine development is antigen degradation by commensal bacteria, proteases, and the acidic stomach environment, it is necessary to protect it and include suitable mucosal adjuvants to enhance antigen bioavailability and recognition by the elements of the gut-associated lymphoid tissues (Hernández et al., 2014). One alternative to address these challenges is antigen encapsulation and among the approaches explored in this regard is the use of nano and microparticles for oral vaccine delivery. There is an array of different polymers suitable for this purpose, which can be either natural or synthetic, in which antigens can be encapsulated within the particles. Although promising, it should be considered that these systems require a synthesis approach that involve strict reaction conditions for synthesis, purification and characterization, which is laborious and costly (Sinyakov et al., 2006).

In contrast, *Schizochytrium* sp. can be used not only as the biofactory of antigens but also as a natural microcapsule (9–14 μm), which is easier and cheaper to obtain than synthetic micro particles. When an antigen is intracellularly accumulated algae biomass can be used as a microencapsulated vaccine not requiring complex processing (i.e., purification). In this manner, the microalgal cell adds its components to the vaccine activity, which could favorably influence: (i) the antigen bioavailability as it is believed to protect the antigen from degradation but at the same time to mediate a proper release of the antigen to make it bioavailable while maintaining its native conformation in the microalgae and therefore the antigenic determinants are preserved (Gregory et al., 2013); and (ii) stimulation of the cells involved in antigen translocation, processing and presentation by the action of algal compounds; improving the response triggered by the antigen. In fact, microalgae have allowed the oral delivery of intact nanobodies in the intestine of mice (Barrera et al., 2015). Therefore, antigens encapsulated into *Schizochytrium* cells offer a cheaper and more practical system compared with conventional micro and nanoparticulated systems.

### Presence of immunostimulatory compounds

Immunostimulatory compounds, such as adjuvants and immunostimulants, can enhance vaccine efficacy since they support the induction of robust immune responses through several mechanisms (Reed et al., 2013, Table 1). The benefits of immunostimulatory compounds include enhanced immunogenicity, antigen-sparing, and achievement of long-lasting immunoprotection (Petrovsky and Aguilar, 2004; Lee and Nguyen, 2015). Therefore, immunostimulatory compounds may contribute to reduce the number and magnitude of antigen doses as well as achieving proper immune polarization (Reed et al., 2013; Ranasinghe, 2014). Using organisms that serve as biofactories but at the same time as delivery vehicles containing immunostimulatory compounds is the ideal case for vaccine design (Rosales-Mendoza and Salazar-González, 2014).

**Table 1:** Compounds produced by *Schizochytrium sp*. with known immunostimulatory activity.

**Compound**	**Immunomodulatory activity and/or mechanism**	**References**
DHA and EPA	Anti-inflammatory response through the inhibition of the kinases JNK and ERK of the NFκβ pathway, which leads to reduced production of cytokines such as TNF-α, IFN-γ, IL-1β, IL-6, IL-12, and enhanced systemic IgG and mucosal IgA production	Xue et al., 2006; Weldon et al., 2007; Draper et al., 2011; Siriwardhana et al., 2012; Magee et al., 2012; Tsunoda et al., 2015; He et al., 2017; Dawczynski et al., 2018
Palmitic acid	Pro-inflammatory response through the TLR4/IKKβ/NFκβ pathway, which leads to increased production of monocyte chemoattractant protein-1, nitric oxide, TNF-α, IL-1β, IL-2, IL-5, IL-6, IL-8, IL-15, and IFN-γ; and enhanced mucosal IgA production	Karsten et al., 1994; Medzhitov et al., 1997; Kim et al., 2007; Nguyen et al., 2007; Schaeffler et al., 2009; Kunisawa et al., 2012; Zhou et al., 2013
Squalene	Squalene-based adjuvants exert a proinflamatory response, which involves the production of cytokines and chemokines, such as MCP-1, IL-1β, IL-8 (CXCL-8), CCL3, CCL4 and IL-4; and enhancement of IgG production	Calabro et al., 2013 Vinay et al., 2013
Polysaccharides	Mechanisms involved in their expected immunomodulatory effects are unknown	Laurienzo, 2010 Chang et al., 2013 Liu et al., 2014

In this context, marine organisms serve as a source of a myriad of potent bioactive compounds; including immunostimulatory molecules of relevance in vaccination. In particular, several algae species have been identified as a source of inflammatory modulators as well as anti-nociceptive and anti-cancer compounds (De Almeida et al., 2011; Farooqi et al., 2014; De Jesus Raposo et al., 2015). To date, the compounds produced by *Schizochytrium sp*. with known anti-nociceptive and anti-cancer effects are DHA and EPA (van Beelen et al., 2007; Mann et al., 2018; Mitome et al., 2018). In addition, *Schizochytrium sp*. contains several bioactive compounds such as flavonoids, β-glucans, β-carotene, polysaccharides, nucleotides and peptides; many of them considered immunostimulants and immunomodulators that, in adequate amounts or in appropriate combination, can improve immune competence (Ibañez and Cifuentes, 2013; Kousoulaki et al., 2015). Interestingly, no signs of toxicity have been observed thus far in the use of bioactive compounds from *Schizochytrium sp*. in humans or animals. Moreover, no intermediary metabolites involved in the synthesis of toxic compounds have been reported in *Schizochytrium sp*. Overall, these features make *Schizochytrium sp*. an attractive species to be used for food purposes and as a valuable source of immunostimulatory compounds for animals and humans (Mioso et al., 2014). The relationship between *Schizochytrium sp*. immunostimulatory compounds and the immune system in the context of vaccine development will be briefly discussed in the following sections.

#### Lipids

Lipids represent up to 56% of the total dry weight of *Schizochytrium* sp. (Yokochi et al., 1998; Ren et al., 2010), with some of them exerting health-promoting effects (Chen et al., 2014b; Raposo and De Morais, 2015). Lipids with immunostimulatory or immunomodulatory activities produced by *Schizochytrium sp*. comprise DHA, EPA, palmitic acid, and squalene (Huang et al., 2010; Taparia et al., 2016). DHA and EPA have proinflammatory and antinflammatory effects (Kelley, 2001; Ramakers et al., 2005) and improve Th1 and Th2 responses following vaccination (Hogenkamp et al., 2011). DHA and EPA immunomodulatory effects have been evaluated *in vitro* in cells derived from human and animals such as macrophages, dendritic cells, neutrophils, lymphocytes, epithelial cell lines, among others. Therefore, DHA and EPA differently modulate immune responses related to phagocytosis, phosphorylation of intracellular signaling molecules, activation of transcription factors, and effector immune-related gene expression; which largely depends of the cell type or target species. The overall modulatory effects of DHA and EPA are related to a polarized anti-inflammatory response demonstrated by the reduction of cytokines such as TNF-α, IFN-γ, IL-1β, IL-6, IL-12, and the anti-inflammatory cytokine IL-10, through the inhibition of kinases (i.e., JNK, ERK) of the NFκβ pathway (Xue et al., 2006; Weldon et al., 2007; Draper et al., 2011; Magee et al., 2012; Siriwardhana et al., 2012; Tsunoda et al., 2015; He et al., 2017; Dawczynski et al., 2018). These effects could be associated with the eicosanoid synthesis pathway (Lokesh et al., 1988). In addition, it should be taken into account that DHA and EPA enhance neutrophil and macrophage phagocytosis, nitric oxide production (a pro-inflammatory mediator), and lymphocyte proliferation (Omura et al., 2001; Verlengia et al., 2004; Gorjão et al., 2006, 2009). Furthermore, it has been suggested that DHA promotes the production of pro-resolving cytokines from T helper lymphocytes and monocytes, via activation of the PPAR-gamma transcription factor; which finally contributes to adequate pro-resolving inflammatory responses that maintain a healthy status (Jaudszus et al., 2013). Moreover, it has been reported that the administration of DHA and/or EPA regulates the immune response in several animals such as cattle, goats, poultry, and pigs (Moreno-Indias et al., 2012; Bragaglio et al., 2015; Swiatkiewicz et al., 2015). Therefore, DHA and EPA could improve the efficacy of vaccines against inflammatory disorders. Interestingly, supplementation with DHA modulated antigen-specific T cell responses through an IL-10-mediated mechanism in vaccinated pigs (Bassaganya-Riera et al., 2007), and reduced TNF-α and IL-1β production and increased IgG titers against bacterial toxins in vaccinated infants (López-Alarcón et al., 2008; Furuhjelm et al., 2011). Similarly, the supplementation of the maternal diet with DHA positively favors the activation of B cells and the response to a potential food antigen upon challenge in suckled offspring (Richard et al., 2015). On this regard, the evidence suggest that both DHA and EPA promote B cell activation and antibody production, particularly enhancing mucosal IgA responses, which is relevant to protect against infectious diseases (Gurzell et al., 2013; Teague et al., 2013; Whelan et al., 2016). Remarkably, the study of DHA and DHA-derivatives as potential adjuvants seems promising in vaccinology. Using 17-HDHA led to an enhanced serum protective antibody response after OVA and H1N1 vaccination in a mouse model (Ramon et al., 2014). Dietary DHA has also been proposed as a potential adjuvant in cancer treatments (Merendino et al., 2013) and has been tested in children and adolescents with acute lymphoblastic leukemia (Elbarbary et al., 2016). It is clear that dietary fatty acids influence the response of the immune system to vaccination and the potential benefits from marine (n-3) PUFA have been reported (Hogenkamp et al., 2011). Therefore, it can be expected that the fatty acids existing in *Schizochytrium*-based vaccines may account for the efficacy of the formulation (Maroufyan et al., 2012).

Another fatty acid present in high levels in *Schizochytrium sp*. is palmitic acid (PA), which triggers a pro-inflammatory response by the activation of macrophages (Talbot et al., 2014; Tian et al., 2015). In addition, PA is involved in the improvement of antigen presentation by antigen presenting cells (APC); an effect that is partly mediated by TLR4 and TLR2 binding (Weatherill et al., 2005; Huang et al., 2012). In general, it is known that PA exerts immunostimulatory effects through the TLR4/IKKβ/NFκβ pathway. The downstream TLR4 signaling induced by PA leads to the activation of NF-kB and it has been associated with an increase in the secretion of monocyte chemoattractant protein-1 and pro-inflammatory molecules, such as nitric oxide, TNF-α, IL-1β, IL-6, and IL-8 (Medzhitov et al., 1997; Kim et al., 2007; Schaeffler et al., 2009; Zhou et al., 2013). In particular, the production of IFN-γ and IL-2 is enhanced in human peripheral lymphocytes upon PA treatment (Karsten et al., 1994). Additionally, PA was found to enhance secretory IgA responses; which are supported by the production of interleukins such as IL-5, IL-6, IL-10, and IL-15 (Nguyen et al., 2007; Kunisawa et al., 2012). On the other hand, dietary administration of PA in mice stimulates plasma cells to produce antibodies in intestine, highlighting its potential as a diet-mucosal adjuvant (Kunisawa et al., 2012). Particularly, PA-diet supplementation induced higher intestinal IgA responses in orally-immunized mice with OVA antigen and cholera toxin (Kunisawa et al., 2014). Interestingly, the palmitoyl group is also crucial in the approaches to produce immunogenic conjugates able to elicit specific and long lasting humoral immune response without the need of additional adjuvants (Kargakis et al., 2007). Vaccine formulations containing PA and palmitic acid-derivatives as adjuvants resulted in improved efficacy against several diseases including tuberculosis (Gupta et al., 2016), cancer (Rueda et al., 2017), rabies (Liu et al., 2016), canine distemper (Chua et al., 2007), and toxoplasmosis (Tan et al., 2010); an effect that is associated with enhanced pro-inflammatory cytokine production (Moyle, 2017).

*Schizochytrium* lipids are also the basis of some commercial adjuvants. Squalene is a lipid (polyunsaturated triterpen) of the terpenoid family, typically obtained from animal sources; however, recent advances in purification processes have allowed the use of plants and microalgae as squalene sources (Brito et al., 2011). Interestingly, squalene is particularly produced at high levels in *Schizochytrium* (Yue and Jiang, 2009; Hoang et al., 2014). Squalene is used to produce adjuvants with proven efficacy, such as MF59, AF03, and AS03 (Reddy and Couvreur, 2009; O'Hagan et al., 2012; Kedl and Kedl, 2015; Bonam et al., 2017). Although the activity of such adjuvants is consequence of the combination of squalene with other compounds, it has been reported that neutrophils, dendritic cells and macrophages are the main players involved in the production of proinflamatory cytokines and chemokines (Calabro et al., 2013). Intraperitoneal administration of pure squalene (536.5 ul kg^−1^) in fish led to safe inflammatory cellular and humoral responses at the site of injection and in immune-relevant tissues (Vinay et al., 2013). It is known that squalene is efficiently absorbed through the intestinal mucosa, rapidly enter into lymphatic circulation and is metabolized (Tilvis and Miettinen, 1983; Gylling and Miettinen, 1994) and therefore it could be active in oral vaccine formulations. For instance, MF59 has been licensed in more than 20 countries for use in an improved influenza vaccine called Fluad® (Frey et al., 2014). Squalene-based adjuvants efficiently enhance immune responses and are safe for humans and animals (O'Hagan et al., 2012; Fox and Haensler, 2013; Black, 2015; Haensler et al., 2015).

Therefore, lipids produced by *Schizochytrium sp*. might account, in combination with other inmunostimulatory molecules synthesized by the alga, for the immunogenicity of vaccines. The current information in the literature encourages performing more studies to investigate in detail the immunostimulatory effects of individual compounds or mixtures of them in oral immunization prototypes. All of this evidence on the production of lipids with immunomodulatory properties accounts for the potential of *Schizochytrium sp*. as an attractive host for the development of efficient oral vaccines.

#### Polysaccharides

Polysaccharides derived from marine microorganisms have had great importance in the industry (Sudha et al., 2014) and are also of relevance for the biomedical field having several applications. For instance, they can serve as vaccine vehicles and adjuvants (Petrovsky and Cooper, 2011; Shinchi et al., 2015). In fact, several polysaccharides have anti-tumor and immunomodulatory properties (Yang and Zhang, 2009; Laurienzo, 2010; Na et al., 2010). In particular, sulfated polysaccharides significantly improved the humoral response; an effect associated to the promotion of lymphocyte proliferation and macrophage activation via TLR-4 binding (Huang et al., 2008). In addition, several polysaccharides favor Th1 responses, promoting protection against intracellular pathogens such as mycobacteria (Pi et al., 2014). A remarkable case is the ADVAX ™ adjuvant, made with pectin, which enhances the humoral and cellular responses against hepatitis and influenza vaccines and is currently under clinical evaluation (Saade et al., 2013; Honda-Okubo et al., 2015).

Marine microalgae are known as one of the most abundant sources of polysaccharides (Laurienzo, 2010). Particularly *Schizochytrium sp*. produces high levels of exopolysaccharides (EPS), at rates of around 300 mg per liter of culture (Chang et al., 2013), that are easily isolated from cultures since these are exported to the culture media (Laurienzo, 2010; Liu et al., 2014). EPS synthetized by the *Schizochytrium* species and other members of the Labyrinthulomycetes class are of great biotechnological interest (Jain et al., 2005). Overall, EPS exert immunostimulatory activity and adjuvant effects (Feng et al., 2015; Li and Wang, 2015), although no report exists about the immunostimulatory activity of EPS from *Schizochytrium sp*. and it constitutes an open field of study. Interestingly, it is known that the EPS production can be modulated by certain factors, such as glucose concentration in culture media; therefore, optimization of the EPS synthesis during *Schizochytrium*–based vaccines production could be an important aspect to optimize (Liu et al., 2014).

*Schizochytrium sp*. also exports many other compounds into the culture media including proteins, lipids, uronic acids, and sulfates (Lee Chang et al., 2014). Moreover, *Schizochytrium sp*. produces high amounts of xanthophylls (Aki et al., 2003) which possess immunomodulatory properties that promote cellular and humoral responses (Park et al., 2011; Ghodratizadeh et al., 2014).

Although no detailed characterization on the cell wall composition is available for *Schizochytrium sp*., it can be expected that the cell wall of *Schizochytrium sp*. could exert singular inmunostimulatory effects. It is known that microalgae possess a singular cell wall composition: an apparent lack of cellulose and the cell wall components are layers of crystalline Ara-rich, Hyp-rich glycoproteins (Miller et al., 1974; Roberts, 1974). Therefore, it can be speculated that the components from the cell wall of *Schizochytrium sp*. may exert inmunostimulatory activity leading to highly effective oral vaccines when used as delivery vehicle.

## Genetic engineering tools

The current genetic engineering methods for *Schizochytrium sp*. comprise transgene installation into the nuclear genome (Table 2), which has been achieved by the following transformation techniques: particle bombardment (Lippmeier et al., 2009; Metz et al., 2009; Sakaguchi et al., 2012; Bayne et al., 2013); *Agrobacterium*-mediated transformation using protoplasts (Cheng et al., 2012); and electroporation (Cheng et al., 2011).

**Table 2:** Summary of the genetic engineering methodologies implemented for the marine microalgae *Schizochytrium* sp.

**Objective**	**Vector**	**Promoter**	**Terminator**	**Transformation method**	**Selection approach**	**Findings**	**References**
Generate mutants for polyunsaturated fatty acid (PUFA) synthase to characterize PUFA biosynthesis	Schizochytrium PFA1: (pBSK:A) Resistance to zeocin: (pTUBZEO11-2)	*Schizochytrium* α-tubulin	Simian virus SV40	Particle bombardment	Zeocin resistance (*Sh ble* gene)	Mutants are auxotrophic and required supplementation with PUFAs Transformation efficiency: 10–100 primary transformants per bombardment	Lippmeier et al., 2009
Generate mutant for fatty acid synthase (FAS) by homologous recombination to characterize fatty acid biosynthesis	(pBluescript SK(þ) from Stratagene)	*Schizochytrium* α-tubulin	SV40	Particle bombardment	Zeocin (*Sh ble* gene)	Mutants are lethal and rescued only when grown under supplementation with appropriate saturated fatty acids in combination with methylated cyclodextrins	Metz et al., 2009
Investigate a transgene expression system by 18S rDNA-targeted homologous recombination	pUCT-18S	TEF1	CYC1	Electroporation	Zeocin (*Sh ble* gene)	The majority of the transformants showed similar biomass and total lipid content when compared to the wild type strain Transformation efficiency: 1 μg linearized plasmid yield more than 100 transformants	Cheng et al., 2011
Develop a novel transformation approach using *Agrobacterium tumefaciens* and a binary vector	pCAMBIA2301	*egfp* gene: TEF1 *gus* gene: CaMV35S	*egfp* gene: CYC1 *gus* gene: nos	*A. tumefaciens*.	G418 resistance (*nptII* gene)	*A. tumefaciens* allowed for the stable insertion of the T-DNA Transformation efficiency: 150 transformants of *Schizochytrium sp*. per experiment	Cheng et al., 2012
Introduce the *Escherichia coli* acetyl-CoA synthetase (ACS) gene to reduce the negative impact of acetate accumulation on the fermentation products	pBluescript II SK (+)	TEF1	CYC1	Electroporation	G418 resistance (*nptII* gene)	The genetically modified *Schizochytrium sp*. showed a significantly higher biomass and fatty acid proportion. Transformants produce lower acetate levels (0.84 and 0.66 g/l) than the wild-type strain (1.66 g/l) Transformation efficiency: unspecified	Yan et al., 2013
Produce the recombinant hemagglutinin (rHA) protein derived from A/Puerto Rico/8/34 (H1N1) influenza Virus as a subunit vaccine	pCL0143, pCL0154, pCL0161, pCL0160, pCL0153	Elongation factor 1	PFA3	Particle bombardment	Paromomycin resistance (*aphH* gene)	Protective immunity against a lethal challenge with homologous virus was achieved by immunizing with a single dose of 1.7, 5, or 15 mg rHA with or without adjuvant (survival rates: 80–100%) Full protection (100%) was achieved at all dose levels with or without adjuvant when mice were given a second vaccination	Bayne et al., 2013
Develop a versatile transformation system for thraustochytrids applicable to both multiple transgene expression and gene targeting	pGEM-T Easy (Promega) and pUC18 (TaKaRa Bio)	EF-1α promoter	Ubiquitin terminator	Particle bombardment and electroporation	G418 resistance (*nptII* gene) Hygromicin resistance (*hyg* gene)	A multiple gene expression and gene targeting was achieved Transformation efficiency Microprojectile bombardment: 4.6 × 10^1^ Colonies/μg DNA vector Electroporation: 0 Colonies	Sakaguchi et al., 2012
Increase the levels of docosahexaenoic acid by the heterologous expression of ω-3 fatty acid desaturase from the nematode *Caenorhabditis elegans*. Achieve site-directed mutagenesis of an acetolactate synthase gene	1. pTUBZEO11-2 2. plasmid pMON50201, or ALSmut1-7; pMON50202, or ALSmut2-2; pMON50203, or ALSmut3-5	*Schizochytrium* tubulin gene promoter	SV40 terminator	Particle bombardment and electroporation	Zeocin (*Sh ble* gene)	Expression of the ω-3 fatty acid desaturase increased the levels of docosahexaenoic acid in *Schizochytrium sp*. Homologous recombination occurs in *Schizochytrium sp*., allowing for the generation of site-directed mutants Transformation efficiency: about 68% of the resulting Zeocin™-resistant clones were PCR positive for the fat-1 gene	http://www.google.com/patents/US7001772

Selective agents used in these successful methods include paromomycin, zeocin and geneticin. In terms of regulatory sequences, the EF1 promoter has led to convenient protein yields up to 5–20 mg of recombinant protein per liter of culture (Cheng et al., 2013). However, no extensive evaluation of distinct regulatory sequences and a wide diversity of target proteins have been explored in *Schizochytrium sp*. Despite the fact that homologous recombination occurs in *Schizochytrium sp*. and it has been exploited to generate mutants for lipid metabolism studies (Metz et al., 2009), this feature has not been exploited to generate clones with site directed insertions to favor efficient expression of the transgene in vaccine production.

In this context, the knowledge on the genetic engineering approaches explored for the case of the *C. reinhardtii* microalga model is a relevant reference that can be used to expand the developments for *Schizochytrium sp*. According to several reports, *C. reinhardtii* tends to show complex expression patterns under nuclear expression approaches (Mardanova et al., 2015), however improvements on yields have been achieved by a number of approaches. For instance, co-expressing the gene of interest and the gene marker in a transcriptional arrangement where, once under translation, the presence of the picornaviral 2A element between both sequences induces the release of independent proteins. The yields obtained under this approach were up to 0.25% TSP. Nonetheless, the limitations of this approach include the fact that this split mechanism does not occur in all the molecules and thus a fraction of the produced protein corresponds to fusion proteins (Rasala and Mayfield, 2015). Another approach has consisted on generating mutant strains by UV light exposure and the use of codon optimized selectable markers (Barahimipour et al., 2016). A bicistronic arrangement driven by the promoter of the endogenous intraflagellar transport 25 protein has led to attractive protein yields (Dong et al., 2017). Insertions of the first intron of the ribulose-1,5-bisphosphate carboxylase/oxygenase small subunit 2 (rbcS2i1) along with codon-optimized coding sequences has proven to enhance the yields of the recombinant protein (Baier et al., 2018).

**Figure 2:**
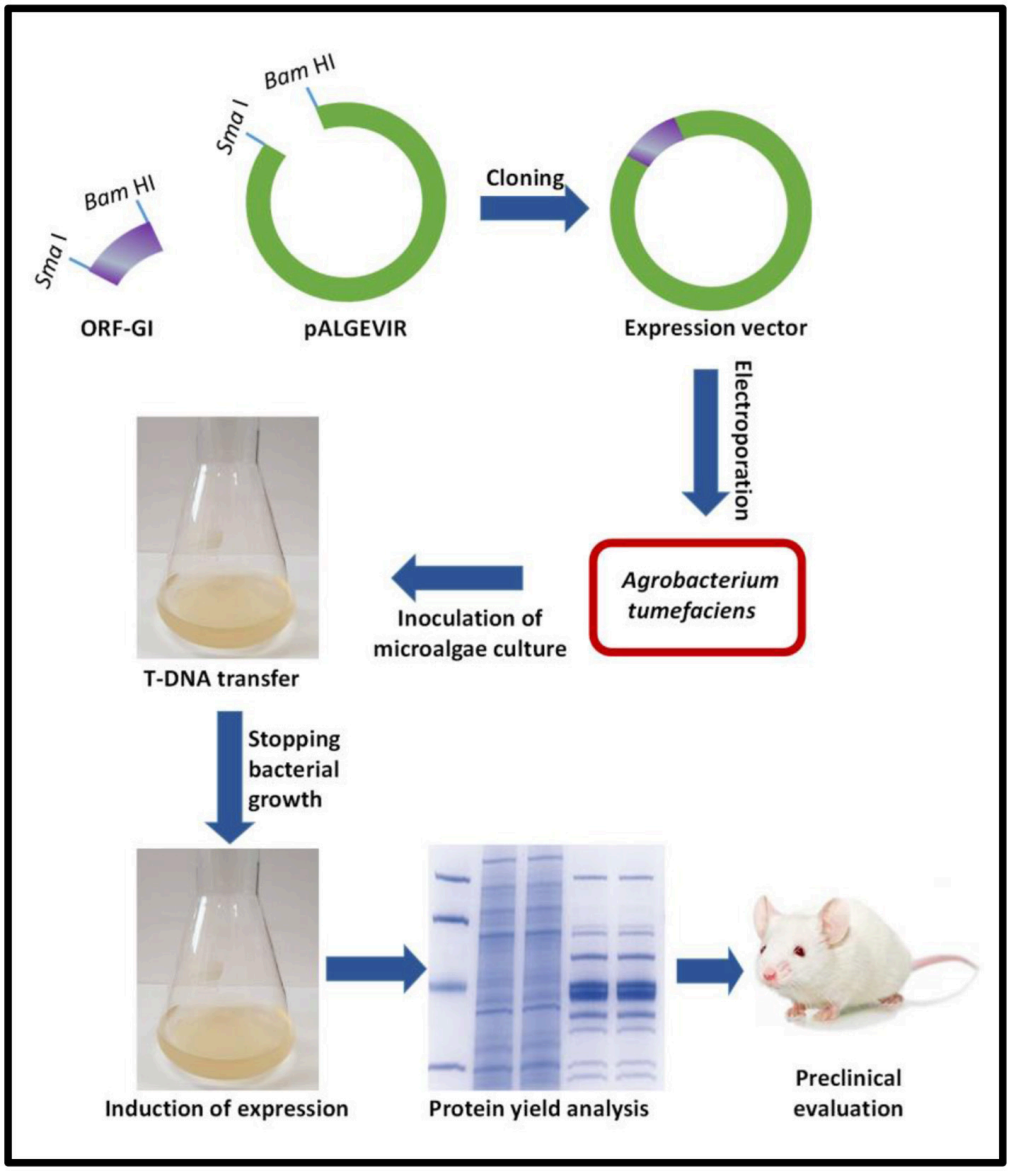
General workflow for the Algevir system. (i) The Open Reading Frame of the Gene of Interest (ORF–GI) is cloned into the pALGEVIR plasmid through the restriction sites *Sma*I and *Bam* HI, (ii) The expression vector is transferred via electroporation to *Agrobacterium tumefaciens*, (iii) Microalgae culture is inoculated with the recombinant *A. tumefaciens* strain carrying the pALGEVIR vector and incubated during 16 h to allow T-DNA transfer, (iv) Agrobacterium growth is stopped by the addition of cefotaxime, (iv) Expression is induced by adding 1% ethanol, (v) biomass is harvested to determine protein yields and perform preclinical evaluation.

Looking to develop innovative expression systems, we have reported for the first time the use of a viral vector to efficiently produce biopharmaceuticals in algae, using *Schizochytrium sp*. as the model alga and a geminiviral vector constructed with sequences of the begomovirus *Ageratum enation virus* (Bañuelos-Hernández et al., 2017). The vector mediates the inducible expression driven by the AlcA promoter, which has a fungal origin and is induced by ethanol in a mechanism mediated by the AlcR protein. The proof of concept was provided by expressing in *Schizochytrium sp*,. a complex viral protein, namely GP1 protein from *Zaire ebolavirus*, and a bacterial toxin subunit (B subunit of the heat-labile *E. coli* enterotoxin). High levels of the target antigens were achieved (GP1 yields up to 1.2 mg per gram of fresh weight biomass). Figure 2 depicts the general methodology comprised by the Algevir system.

In terms of yields, *Schizochytrium sp*. possesses a competitive productivity for recombinant protein production (5–20 mg/L) when compared with other microalgae. For instance, maximum yields observed in photosynthetic algae (*C. reinhardtii*) are 15 mg/l in a system based in the secretion of the target protein using either synthetic glycomodules (tandem serine and proline repeats) that improve secretion or a mutant strain that efficiently expresses heterologous genes (Bayne et al., 2013; Lauersen et al., 2013; Ramos-Martinez et al., 2017). Another promising approach relies in chloroplast genome engineering, by which yields up to 3 mg/L have been achieved (Gimpel et al., 2015). However *Schizochytrium sp*. does not possesses this organelle.

In this context, Algevir is a robust and attractive system as it possesses the following advantages that override the mentioned limitations: nuclear expression offers the possibility to access the complex cellular machinery to perform complex post-translational modifications (e.g., glycosylation); intracellular accumulation at high levels of the biopharmaceutical allows using the alga cell as the delivery vehicle in oral formulations; inducible expression allows a tight control that might lead to an efficient production of recombinant proteins that have toxic effects in algae; transient expression avoids the long time investment required to select stably transformed clones. Therefore, Algevir is a versatile system offering the advantages of transient expression (short production time and high yields) that are ideal for the production of vaccines in response to epidemics.

Conventional systems for recombinant protein production allow overall higher yields than those of the microalgae-based systems. For instance bacterial systems have a productivity in the order of 5 g/l, yeast systems in the order of 30 g/l, whereas mammalian cells are in the range of 5–25 g/l (Jarvis, 2008; Demain and Vaishnav, 2009). However, besides yields it should also be considered that bacterial systems often led to significant losses when refolding is required, the system has limitations for the synthesis of complex proteins and the host produces endotoxins (Feng et al., 2012). In the case of mammalian cells, the limitations are high production cost and possible contamination with human pathogens (Moody et al., 2011). Although yeast is also a fast-growing, GRAS, heterotrophic eukaryotic microorganism leading to high yields of recombinant proteins, it is often associated with hyper- glycosylation problems that may impair correct protein folding and functionality (Wildt and Gerngross, 2005). Therefore, although *Schizochytrium sp*. offers modest productivity in this context, the overall features of this organism make it an attractive host for vaccine production.

## Initial efforts using *Schizochytrium sp*. in vaccine development

Bayne et al. (2013) reported a pioneering study on the expression of hemagglutinin (rHA) from A/Puerto Rico/8/34 (H1N1) influenza Virus in *Schizochytrium sp*. The *Schizochytrium sp*. EF-1 promoter, PFA3 terminator, and the aphH gene conferrying paromomycin resistance were used. Clones transformed by particle bombardment were obtained and characterized at the molecular and immunogenic level. The algae-made HA was successfully detected in the extracellular space retaining activity as revealed by hemagglutination activities from 16 to 512 hemagglutination activity units (HAU)/50 μL of cell-free supernatants (CFS).

The algae-made HA antigen was purified from fermentation culture supernatants with average yields of 5–20 mg of HA per liter of culture. The authors explored several immunization schemes in BALB/c mice groups comprising doses of 1.7, 5, and 15 μg of HA alone or co-administered with the AddaVax^TM^ adjuvant. Another variable was a boost administered 3 weeks after priming. The hemagglutination inhibition (HI) activity assays revealed that the adjuvant-formulated vaccine induced higher HA antibody titers, which significantly increased after the second injection.

The protective immunity against a lethal challenge with a homologous virus was evaluated following an immunization scheme comprising three rHA dose levels (1.7, 5, or 15 μg) with or without adjuvant. In spite of the dose, all mice were fully protected after two vaccinations. In terms of infectious virus titers, the test animals receiving adjuvant showed lower virus titers than adjuvant-free vaccine-treated mice.

The Algevir system has been applied to produce several antigenic proteins with distinct yields, namely the GP1 antigen Zaire ebolavirus (yields: 6 mg/l), the B subunit of the heat labile *E. coli* enterotoxin (yields: 0.4 mg/l), and a chimeric protein targeting the receptor of advanced glycation end products for Alzheimer's disease (RAGE; yields: 0.4 mg/l) (Bañuelos-Hernández et al., 2017; Ortega-Berlanga et al., 2018).

These seminal reports indicate a high potential of *Schizochytrium sp*. in the microalgae-made vaccines field, in particular for the production of antigens requiring complex post-translational modifications in a robust system. Several directions in which this technology can be exploited are identified and discussed in the next section.

## Future directions

The positive outcomes derived from the Influenza vaccine prototype developed with *Schizochytrium sp*. indicate a great potential to develop other vaccine candidates. Several directions in which this organism can be exploited in vaccinology are identified. For instance, biomass from transgenic lines expressing the antigen of interest will allow evaluating oral vaccines formulated in a straightforward manner without complex processing (e.g., freeze-dried biomass could be used for oral immunization). In addition, several cellular localizations can be assessed and the implications on immunogenicity and yields determined. For example, proteins can be retained in the endoplasmic reticulum or expressed in the form of amylosomes as has been performed in *C. reinhardtii* (Dauvillée et al., 2010); another possibility is the association to lipid bodies or protein bodies which have been accomplished in seed crops. In plants, the oil body fusion technology consists in fusing the target protein to the N- or C-terminus of oleosin in the oil body surface; since the expression is driven by a seed specific promoter, the protein can be efficiently expressed and rescued from seeds (Stoger et al., 2005; Boothe et al., 2010). This approach also extends protein half-life allowing easier transportation and storage (Bhatla et al., 2010). Given the high accumulation of oil bodies in *Schizochytrium sp*. (Morita et al., 2006), this process is considered viable and proposed as an efficient approach for recombinant antigen production having implications in the immunogenic activity since high molecular size complexes carrying the antigen could be produced in the recombinant algae.

A number of mechanisms may underline the immunomodulatory (pro- and anti-inflammatory) effects of fatty acids present in *Schizochytrium sp*. in prototype vaccines. All of them should be considered case-by-case determining the immunological outcomes required to fight the target disease. Thus, efforts to elucidate the immunological role and impact of the compounds present in *Schizochytrium* sp. are needed to understand and ultimately manipulate the immune responses induced by the vaccine. In terms of antigen design, studying the potential to produce virus like particles, antibody-antigen immunocomplexes, and adjuvant-antigen fusions are relevant, pending goals (Wen et al., 2016; Ding et al., 2018).

Moreover, conventional mutagenesis and genetic engineering along with the currently available *Schizochytrium* sp. genome bring opportunities for implementing strategies to improve the production of recombinant subunit vaccines. For instance, endogenous promoters and signal peptides could be used to improve the expression of the target antigens (Molino et al., 2018). Moreover, modifying the expression of enzymes involved in the synthesis of immunostimulatory compounds could lead to strains with modified metabolite profiles that could lead to improved immunogenicity (Park et al., 2018). Exploring these avenues will reinforce the use of *Schizochytrium sp*. as a convenient host in the production of attractive vaccines. The use of *Schizochytrium sp*. for vaccine production will also require the implementation of Good Management Practices-compliant process and validation of its safety for consumption by humans. It should be considered that *Schizochytrium sp*. is safe as food supplement for animals and thus the veterinary field could be the first to be benefited from the evaluation and commercialization of the *Schizochytrium*-made vaccines (Franklin et al., 1999; Meale et al., 2014; Kousoulaki et al., 2015; Park et al., 2015). This will be highly convenient since aquaculture and poultry intensive farming practices encourage the spread of diseases but the small size and low value of juvenile animals make other vaccination approaches impractical (Charoonnart et al., 2018).

## Conclusions

Safe consumption, bioencapsulation effects, and the presence of immunomodulatory compounds are attributes that account for the potential of *Schizochytrium sp*. as an innovative platform for oral vaccine development. In addition, the well-established industrial process for *Schizochytrium sp*. production and the availability of efficient genetic engineering tools will support the perspectives of this technology.

Therefore, *Schizochytrium sp*. is an interesting alga species with implications in the development of improved algae-made vaccines that will benefit animal and human health. This research path will be particularly relevant in developing countries where heat-stable, low cost, easy to administer, and safe vaccines are urgently needed.

## Author contributions

CA and SR-M conceived the manuscript. AR-V wrote most of the sections under supervision of SR-M and CA. BB-H wrote the section on the Algevir system.

### Conflict of interest statement

The authors declare that the research was conducted in the absence of any commercial or financial relationships that could be construed as a potential conflict of interest.
